# Secondary Dislocations in Type B and C Injuries of the Subaxial Cervical Spine: Risk Factors and Treatment

**DOI:** 10.3390/jcm13030700

**Published:** 2024-01-25

**Authors:** Philipp Raisch, Jan Pflästerer, Michael Kreinest, Sven Y. Vetter, Paul A. Grützner, Matthias K. Jung

**Affiliations:** Department for Trauma and Orthopaedic Surgery, University of Heidelberg, BG Klinik Ludwigshafen, Ludwig-Guttmann-Strasse 13, 67071 Ludwigshafen, Germany; philipp.raisch@bgu-ludwigshafen.de (P.R.); jan.pflaesterer@bgu-ludwigshafen.de (J.P.); michael.kreinest@bgu-ludwigshafen.de (M.K.); sven.vetter@bgu-ludwigshafen.de (S.Y.V.); paul.gruetzner@bgu-ludwigshafen.de (P.A.G.)

**Keywords:** cervical spine fracture, secondary dislocation, instability, adverse events, stabilization surgery

## Abstract

Introduction: This study analyzed the incidence of secondary dislocations (sDLs) after surgical stabilization of AO Spine type B and C injuries of the subaxial cervical spine (sCS). Materials and Methods: Patients treated for injuries of the sCS from 2010 to 2020 were retrospectively analyzed for the incidence of sDL within 60 days after first surgery. A univariate analysis of variables potentially influencing the risk of sDL was performed. Patients with solitary anterior stabilization underwent subgroup analysis. The treatment of sDLs was described. Results: A total of 275 patients were included. sDLs occurred in 4.0% of patients (*n* = 11) in the total sample, most frequently after solitary anterior stabilization with 8.0% (*n* = 10, *p* = 0.010). Only one sDL occurred after combined stabilization and no sDLs after posterior stabilization. In the total sample and the anterior subgroup, variables significantly associated with sDL were older age (*p* = 0.001) and concomitant unstable facet joint injury (*p* = 0.020). No neurological deterioration occurred due to sDL and most patients were treated with added posterior stabilization. sDL is frequent after solitary anterior stabilization and rare after posterior or combined stabilization. Discussion: Patients of higher age and with unstable facet joint injuries should be followed up diligently to detect sDLs in time. Neurological deterioration does not regularly occur due to sDL, and most patients can be treated with added posterior stabilization.

## 1. Introduction

Acute discoligamentous injuries type B and type C of the subaxial cervical spine (sCS), according to the AO Spine Subaxial Injury Classification System [1], regularly necessitate surgical intervention with primary treatment goals being decompression of neural structures and restoring physiological alignment as well as stability [2,3,4]. Standard means of stabilization after reduction are anterior plate fixation of injured spine segments, often with discectomy and intervertebral fusion (ACDF), and posterior pedicle or lateral mass screw placement and internal rod fixation.

Treatment failure in the form of instability and secondary dislocation (sDL) after initial stabilization is reported in 5–13% of cases [5,6,7,8] and carries a risk of spinal cord and nerve root injury and pain. Self-evidently, the secondary dislocation rate (sDLR) depends on various patient-, injury-, and treatment-related factors. Considerations relating to stability must be weighed against other factors influencing surgical approach like access to the compressed spinal cord or locked facet joints, and to the risks for specific complications after anterior or posterior surgery [9,10]. Other relevant aspects may be the access to concomitant injuries, or circulatory or pulmonary instability in multiple trauma [11] as a contraindication to prone positioning on the operating table.

Due to this complex and time-critical decision-making process, it is essential to know the incidences and risk factors for instability and sDLs in the injured sCS. While the influence of stabilization seems clear, with posterior stabilization being known to yield higher biomechanical stability [12], other patient-, injury-, and treatment-related factors need further investigation. Treatment options for sDLs and the rate of neurological deterioration due to sDL must be further characterized in order to effectively evaluate the risk of potential instability when planning the surgical treatment and follow up of patients with sCS injury.

This study aimed to determine the incidence of sDL after primary stabilization surgery of type B and type C injuries of the sCS and to identify patient-, injury-, and treatment-related risk factors for sDLs. Potential neurological deterioration due to sDL and treatment options should be described in order to classify the risk of sDL in clinical decision making.

## 2. Materials and Methods

This was a monocenter case–control study conducted at a national level I trauma center. It was approved by the ethics committee in charge (Ethics Committee of the State Medical Association Rhineland-Palatinate, Mainz, Germany; application number 2021-15816). The STROBE statement was followed for the reporting of this study.

### 2.1. Inclusion and Exclusion Criteria

The inclusion criteria were as follows: (i) stabilization surgery of an acute injury of the sCS type B or C, according to the AO Spine Subaxial Injury Classification System [1] at the study clinic from 2010 to 2020, (ii) preoperative CT scan available, and (iii) no previous surgery of the subaxial cervical spine. Patients were excluded if they were transferred to a different hospital or died within 14 days after surgery and had not experienced a sDL up to that point.

### 2.2. Treatment

Stabilization surgeries were performed as follows: (i) A posterior approach with pedicle or lateral mass screw placement and internal rod fixation. (ii) An anterior approach with fixation via plate, which frequently occurred with discectomy (ACDF). In cases of discectomy, an autologous iliac crest craft or allogenous bone graft was used up to 2015, which were steadily replaced by intervertebral cages, which were exclusively used from 2018 onward. (iii) A primarily planned combined anterior and posterior stabilization, in one session or as a two-stage surgery. The choice of treatment and post treatment was made based on national and international guidelines [2,3,4] by the treating surgeon in charge, and if possible, in accordance with the patient or their relatives. While, if appropriate, anterior stabilization was performed as a standard procedure at the study site, various factors were considered as indications for a posterior or combined stabilization, most notably stiffening spine disease and higher age with reduced bone quality. Besides injury morphology and bone quality, spinal cord compression, pre-existing conditions of the spine, concomitant injuries, and patients’ cardiopulmonary stability were factors taken into consideration when choosing a treatment plan. All patients were recommended radiological follow ups until at least six weeks post operation to detect secondary dislocation or instability if they were discharged prior to that point.

### 2.3. Data Extraction and Variables

Baseline demographic data as well as data on pre-existing spinal conditions were collected. All patients’ injuries were classified according to the AO Spine Subaxial Injury Classification System [1]. We also determined the AO Spine Modifiers and stratified facet injuries according to suspected instability, as postulated in the classification (Table 1).

The primary surgical stabilization (anterior, posterior, combined) for each patient was documented. Here, we deduced the “intention to treat”, meaning that if a primary solitary anterior or posterior stabilization was intended and sDL necessitated escalation to a combined anterior–posterior stabilization, this was counted as revision surgery.

### 2.4. Endpoint and Statistical Analysis

The endpoint for this study was the need for revision surgery because of sDL within 60 days of first surgery. This duration was chosen to include any revisions that were indicated based on radiological abnormalities in the recommended follow up six weeks post operation. Patients were divided into a group with and a group without revision for sDL. Variables that could potentially influence the risk of sDL underwent a univariate analysis. We performed a subgroup analysis of patients with primary solitary anterior stabilization. Demographic variables and variables of injury morphology in patients in the anterior, posterior, and combined treatment groups also underwent univariate analysis to make possible sources of selection bias concerning treatment transparent.

The statistical tests used for the ever variable are stated in the Section 3. Statistical significance was assumed for *p*-values < 0.05. We used the software SPSS for Windows (Version 27, IBM Corp., Armonk, NY, USA).

## 3. Results

### 3.1. Patient Characteristics

**Figure 1:**
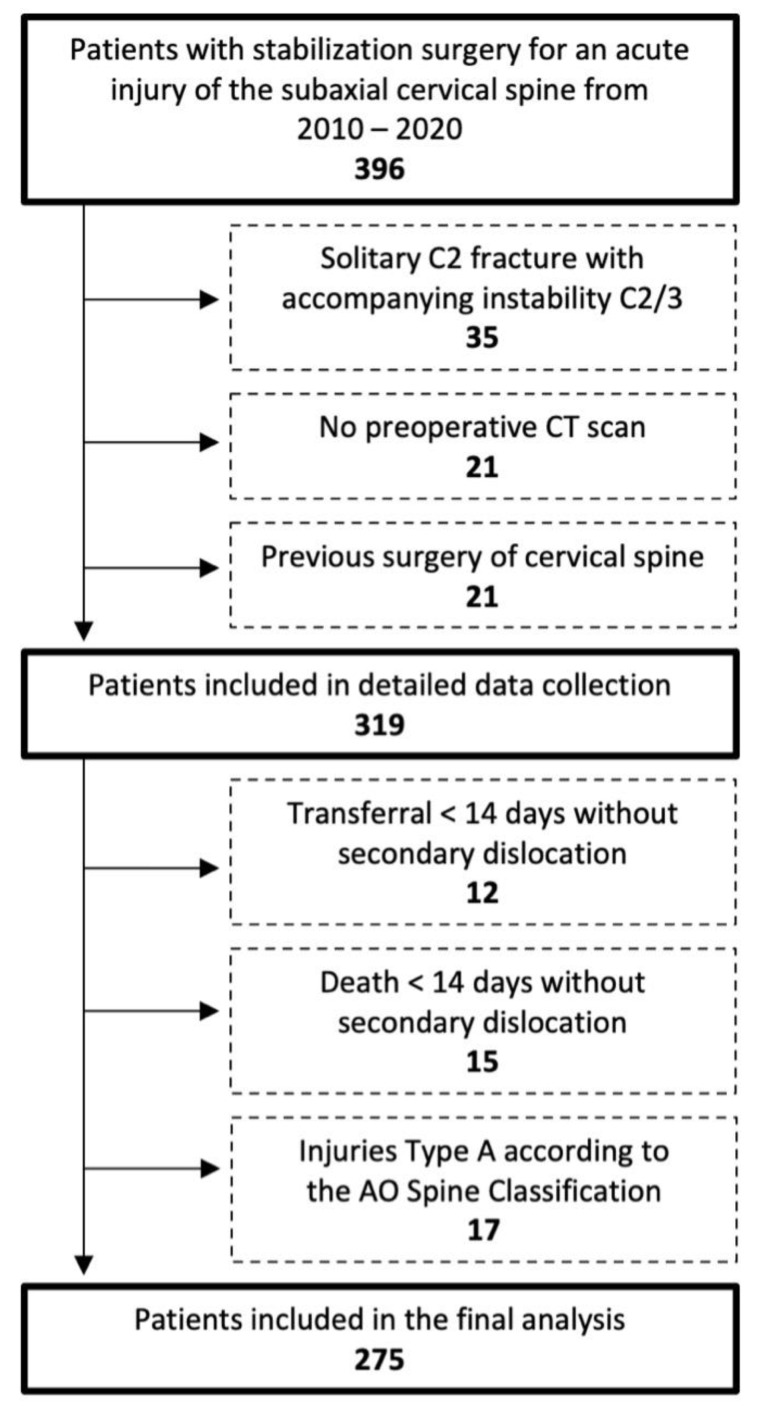
Flow chart of patient inclusion according to inclusion and exclusion criteria.

In total, 275 patients were included in the final analysis (Figure 1). The mean age was 59.3 years (12–91 years, SD 19.9). Of these patients, 26.5% (*n* = 73) were female (Table 2). AO Spine type B2 injuries were present in 14.2% (*n* = 39), type B3 in 34.9% (*n* = 96), and type C in 50.9% of patients (*n* = 140). A multilevel injury was present in 6.5% (*n* = 18) and potentially unstable or unstable facet injury types F2-F4 in 56.4% of patients (*n* = 155). The median follow up was 83 days (6-1923).

Demographic and injury characteristics of patients in the primary anterior, posterior, and combined treatment groups underwent a univariate analysis. Patients in the posterior treatment group were significantly older (mean 70.1 years, SD 16.6) than patients in the anterior (mean 57.4 years, SD 19.8) and combined group (mean 57.3 years, SD 20.0, *p* < 0.001). Also, the prevalence of preexisting stiffening spine pathologies (corresponding to Modifier M3) was significantly different (*p* = 0.023), being most frequent in the posterior treatment group (32.6%), less frequent in the combined (14.0%), and least frequent in the anterior treatment group (4.8%). Various variables of injury morphology according to AO Spine were also distributed significantly differently in the treatment groups. Details on demographic variables, injury morphology, and treatment of different injury types in different age groups are provided in Supplementary Tables S1 and S2.

### 3.2. Primary Stabilization and Secondary Dislocations

Primarily intended solitary anterior stabilization was performed in 45.5% of patients (*n* = 125), solitary posterior stabilization in 15.6% (*n* = 43), and combined stabilization in 38.9% (*n* = 107, Table 3). sDLs occurred in 4.0% of patients (*n* = 11) in the total sample. sDLs occurred most frequently after solitary anterior stabilization (8.0%), most pronounced in type C (13.3%) and type B2 injuries (12.5%), and less frequently in type B3 injuries (3.1%). Only one sDL occurred after combined stabilization and no sDLs occurred after posterior stabilization. The time from surgery to detection of sDL was 3 to 45 days (median 10 days, mean 16 days).

### 3.3. Potential Risk Factors for Secondary Dislocation

Univariate analyses of patient, injury, and treatment characteristics that may potentially influence the risk of sDL showed that patients with sDLs were significantly older (*p* = 0.001), had potentially unstable or unstable concomitant facet joint injuries (*p* = 0.020), and had undergone a solitary anterior stabilization significantly more often (*p* = 0.010, Table 4). Trends were seen toward higher sDLR in female patients (*p* = 0.134) and in patients with pre-existing stiffening spine disease, corresponding to the Modifier M3 (*p* = 0.121).

A subgroup analysis of the 125 patients with primary solitary anterior stabilization, in which one sDL occurred, was performed (Table 5). In this patient group, higher age (*p* = 0.002) and potentially unstable or unstable facet injury (*p* = 0.005) were also significantly associated with sDLs, while trends concerning sex (*p* = 0.088) and stiffening spine disease (*p* = 0.073) were present. There was also a trend towards a higher sDLR if bone grafting (autologous or allogenous) was used after discectomy (*p* = 0.183). Accordingly, the sDLR after anterior stabilization with bone grafting was 11.6% of patients (*n* = 8/69) compared to 3.6% after anterior stabilization with intervertebral cage(s) or without discectomy (*n* = 2/56).

### 3.4. Treatment of Secondary Dislocations

**Figure 2:**
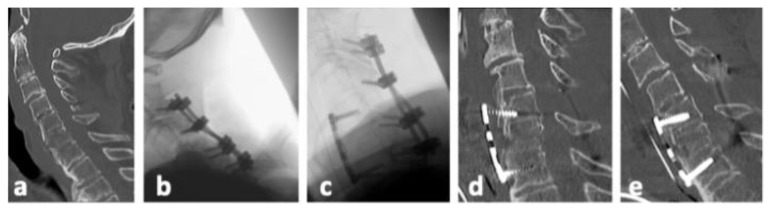
Representative CT images of patient no. 3 (Table 6). (**a**). Postinjury midline sagittal CT reconstruction. (**b**). Posterior stabilization from C3/4 to C6/7 with laminectomy of C5 due to spinal stenosis was performed. (**c**). Four days later anterior plate fixation C5 to C7 was added; no screws were placed in the fractured vertebral body of C6. (**d**). Secondary dislocation was detected in a CT scan 13 days after additional anterior fixation with loosening of posterior screws (not shown) and displacement of the anterior plate. (**e**). Combined revision was performed with posterior stabilization of C3/4/5/6/7 and anterior plate fixation C5 to C7.

**Figure 3:**
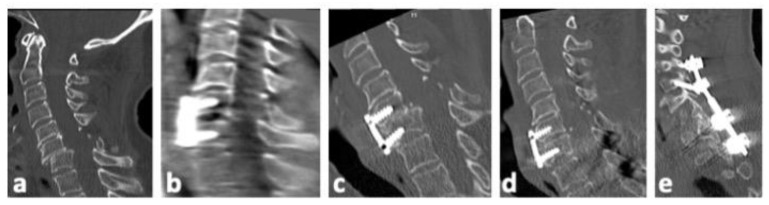
Representative CT images of patient no. 6 (Table 6). (**a**). Postinjury midline sagittal CT reconstruction. (**b**). Intraoperative 3D scan after anterior open reduction, discectomy, and fixation with intervertebral allogenous bone graft and plate C6/7. (**c**). Secondary dislocation of C6/7 nine days post operation. (**d**,**e**). Additional posterior reduction and stabilization of C4/5 to Th1/2 was performed.

**Figure 4:**
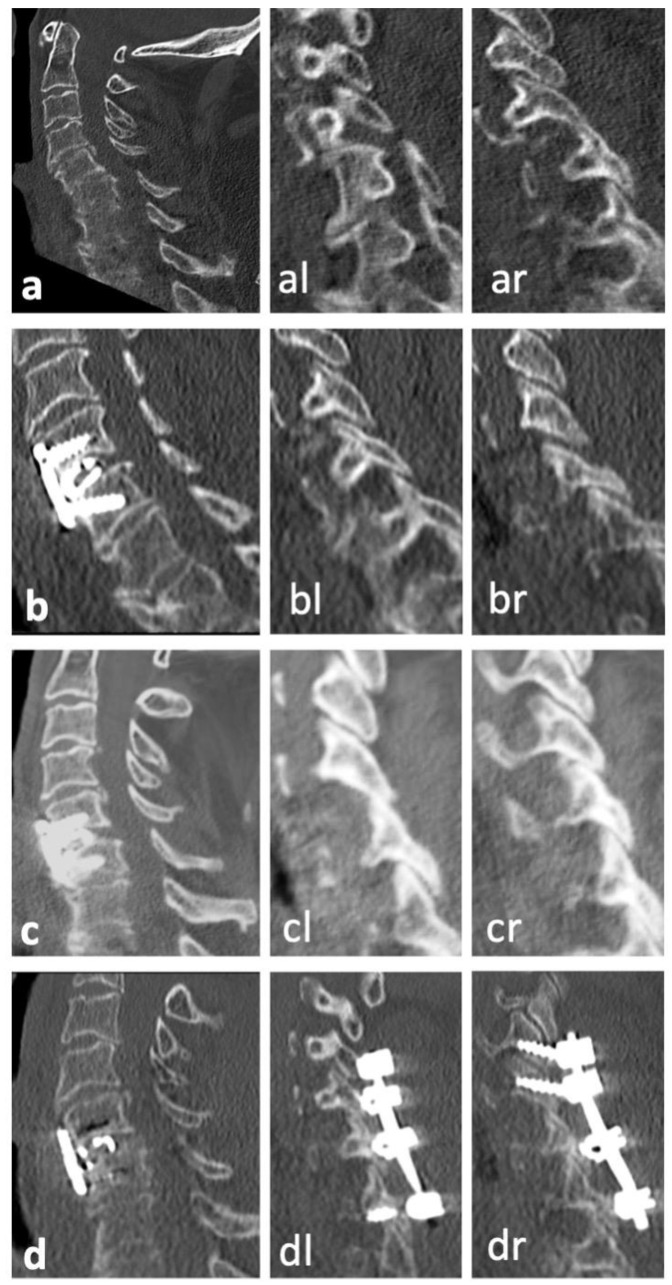
Representative CT images of patient no. 11 (Table 6). (**a**)**.** Postinjury CT scan with midline sagittal reconstruction as well as perched left facet joint (**al**) and subluxation of right facet joint (**ar**) in parasagittal reconstructions. (**b**,**bl**,**br**). Corresponding CT reconstructions after anterior discectomy, intervertebral cage, and plate fixation C6/7 with adequate reduction in the anterior column and right facet joint (**br**) but persisting slight subluxation of the left facet joint (**bl**). (**c**,**cl**,**cr**). The patient presented with persisting neck pain five weeks post operation. CT reconstructions showed secondary dislocation with instability of the anterior column as well as increased subluxation of the left facet (**cl**). (**d**,**dl**,**dr**). Situation after posterior open reduction and additional posterior stabilization C4/5/6/7. While anatomical reposition of the anterior column was not achieved, stability was increased leading to pain relief and no further dislocation upon follow ups.

There was no neurological deterioration following sDL or its revision surgery in our patient cohort. Table 6 gives details on the eleven patients with sDLs and their further treatments. Most patients with sDLs after anterior stabilization could be treated with added posterior stabilization. One patient with a previous monosegmental anterior stabilization received an anterior revision with bisegmental stabilization, while one patient with a previous combined stabilization received a replacement of anterior and posterior stabilization, including one additional vertebra in the posterior stabilization. Figure 2, Figure 3 and Figure 4 show representative CT scans of three patients with sDLs.

## 4. Discussion

### 4.1. Incidence of Secondary Dislocations

In this case–control study of patients with surgery for unstable sCS injuries, revision for sDLs occurred in 4.0% of all patients and in 8.0% after solitary anterior stabilization. Other studies on the treatment of unstable sCS injuries report rates of secondary instability and dislocation of 5–8% across all stabilizations [5,6] and 8–13% for subgroups with solitary anterior stabilization [7,8]. However, the heterogeneity of patient populations, definitions of instability and dislocation, and of treatment modalities limits comparability.

### 4.2. Risk Factors and Patient Characteristics

In our sample, patients with sDLs were significantly older than those without sDLs. Age is an independent risk factor for adverse events after spine surgery in general [13]. Considering stability specifically, older age is also associated with lower bone density, which has been shown to be relevant for primary stability in anterior [14] and posterior [15] cervical fixation surgery. The trend toward a higher rate of sDLs in our female patients may have also been due to lower bone density compared to our male patients. To our knowledge, there are no clinical studies directly evaluating dislocation risk depending on patients’ bone density. In the view of a growing number of elderly patients with cervical spine injuries around the world [11,16], this seems to be a relevant question for further research.

We observed a trend towards a higher sDLR in patients with stiffening spine pathologies such as ankylosing spondylitis or ossification of the ligamentum flavum, subsumed under the modifier M3 in the AO Spine Classification [1]. The higher potential for instability in these patients is ascribed to longer levers around the injured segment. This is acknowledged in clinical studies and guidelines, where long posterior or combined stabilization constructs are recommended [2,3,17].

### 4.3. Injury Morphology

Across all stabilization modalities, the sDLR varied nonsignificantly depending on primary injury morphology (B2, B3, C). However, the sDLR after solitary anterior stabilization was markedly lower in B3 injuries, compared to B2 and C injuries. This is plausible, as per definition, posterior stabilizing structures remain intact in B3 injuries.

In contrast to primary injury types, there was a significant association of sDLs with potentially unstable or unstable concomitant facet joint injuries. This is in accordance with biomechanical findings which demonstrate increased instability in injured cervical segments in cases of relevant concomitant injuries to facet joints [18,19] and underlines the importance of taking facet joint injury morphology into consideration when planning surgical stabilization and follow ups. Our findings also support the postulated increasing instability of different facet joint injuries, as stratified by the AO Spine Classification [1].

### 4.4. Treatment

Since the above-mentioned risk factors for sDLs are not modifiable, great importance lies in the choice of approach for primary stabilization. All but one sDL in our cohort occurred after solitary anterior stabilization, which was statistically significant. Within this subgroup, the same variables of higher age and unstable or potentially unstable facet injury were significantly associated with sDLs.

For the aim of achieving high primary stability in an experimental biomechanical setting, posterior stabilization is known to be superior [12]. However, the clinical relevance of these findings is frequently questioned, as some authors argue that anterior stabilization alone returns an injured segment to at least its preinjury stability [9]. Thus, the choice of primary approach is more complex, taking several distinct advantages and disadvantages of posterior and anterior stabilization beyond biomechanical stability into consideration.

Advantages of the anterior approach, especially in trauma, include the supine patient position, direct access to herniated discs, and less surgical trauma, blood loss, and wound complications [10]. Thus, the anterior approach is referred to as the standard approach to the injured sCS in multiple guidelines [2,3,4]. It is also relevant to put the consequences of potential sDLs as a result of solitary anterior stabilization into perspective. Among the eleven cases with sDLs in our cohort, no spinal cord compression or neurological deterioration was seen as a result of sDL. In most cases, adequate treatment could be delivered by adding posterior stabilization without a revision through the anterior approach. There are data showing that combined stabilization does not negatively impact patient reported outcomes compared to solitary anterior stabilization [20]. Thus, for many cases, it seems feasible to choose a solitary primary anterior approach, perform diligent postoperative follow ups, and escalate to stabilization in cases of instability. Additionally, we saw a lower sDLR after ACDF with intervertebral cages compared to bone grafts. This poses the question of higher stability in these constructs. However, investigating this in an experimental setting seems of limited clinical relevance, since other clear advantages of intervertebral cages such as no donor site morbidity, less blood loss, and reduced operating time, as well as equal stability and fusion rates [21,22,23], lead to bone grafting widely being replaced by cages in ACDF.

Finally, regarding post treatment, there are no clinical studies evaluating the use of cervical collars after cervical spine trauma to prevent sDLs. While at our site soft collars are prescribed especially frequently, with rigid collars being reserved for patients with especially poor bone quality, there is no homogenous standard of care at German spine centers [24], and guidelines make no definitive recommendations [2,3,4]. In retrospective analyses, the variable of collar use is subject to great bias, as surgeons might opt for collar use in cases with suspected greater instability in the first place. Considering the known risks of cervical collars, such as pain and pressure ulcers [25], clinical evidence supporting or contradicting their use to prevent sDLs is desirable.

### 4.5. Limitations

This study was limited by its retrospective design and associated forms of bias, especially concerning treatment decisions for surgical stabilization, post treatment, and collar use. Guidelines still leave much room for surgeons’ individual evaluation in treating specific injuries [2,3,4] and many variables must be taken into consideration, as elaborated above. Accordingly, treatment groups in our cohort are heterogenous, with patients with posterior stabilization being significantly older, most likely due to the fact that patients with stiffening spine disease, which increases in prevalence with advancing age, were treated with posterior instrumentation more frequently. However, in a retrospective design, some of these variables and evaluations influencing treatment decisions are not reconstructable and their influences not evaluable. We consider this the study’s most important weakness and strongly advocate for further prospective investigations of this research question.

This study’s primary endpoint, revision surgery for sDL in the phase of acute care, is well documented and clinically significant. However, some cases of sDLs might have been missed due to insufficient follow up. We point out that radiological controls to detect instability were recommended for all patients until at least six weeks post operation. Although practitioners would usually refer patients with abnormal findings to our clinic, it cannot, however, be ruled out that revision surgery might have been performed at a different site.

As this is an exploratory analysis of potential risk factors, no adjustment for multiple testing was performed. Due to the low number of observations of the primary endpoint, no regression model could be established. As a result, we were limited to univariate analyses. Some potential risk factors such as bone density or surgeons’ experience were not retrospectively available; others like the quality of performed procedures were not objectively evaluable and could thus not be analyzed. Finally, heterogeneity in our patient sample must also be pointed out: We included a wide spectrum of patients treated for type B and C injuries of the subaxial cervical spine, comprising various age groups, patients with stiffening bone disease, and different surgical approaches.

## 5. Conclusions

Secondary dislocation (sDL) after surgical stabilization of type B and C injuries of the subaxial cervical spine is a relevant problem. Nonmodifiable factors such as age and concomitant unstable facet injury seem to increase the risk of sDL. Posterior or combined stabilization carries a markedly lower risk of sDL compared to a solitary anterior approach. However, if detected in time, sDL does not regularly lead to neurological deterioration and surgical revision with escalation of stabilization surgery is feasible. Even if viewed from a stability-focused perspective, the anterior approach to the subaxial cervical spine is therefore viable. It should, however, be followed up diligently, especially in older patients and patients with unstable facet injury.

## Figures and Tables

**Table 1:** AO Spine Subaxial Injury Classification: Definitions of Modifiers and Facet Injuries [1].

**AO Spine Modifiers**		
M1	Posterior Capsuloligamentous Complex injury without complete disruption	
M2	Critical disc herniation	
M3	Stiffening/metabolic bone disease (i.e., DISH, AS, OPLL, OLF)	
M4	Vertebral artery abnormality	
**AO Spine Facet Injuries**		Stability
F1	Nondisplaced facet fracture with fragment < 1 cm in height, <40% of lateral mass	Stable
F2	Facet fracture with potential for instability with fragment > 1 cm, >40% lateral mass, or displaced	Potentially unstable
F3	Floating lateral mass	Unstable
F4	Pathologic subluxation or perched/dislocated facet	

AS, ancylosing spondylitis; DISH, diffuse idiopathic skeletal hyperostosis; OLF, ossification of ligamentum flavum; OPLL, ossification of posterior longitudinal ligament.

**Table 2:** Patient demographics and injury morphology [*n*, %].

Demographics		
Age		
≤39	49	17.8%
40–59	75	27.3%
60–79	111	40.4%
≥80	40	14.5%
Sex		
Female	73	26.5%
Male	202	73.5%
Preexisting stiffening spine pathology		
No	240	87.3%
Yes	35	12.7%
Injury morphology		
AO Spine Primary		
B2	39	14.2%
B3	96	34.9%
C	140	50.9%
Multilevel Injury		
No	257	93.5%
Yes	18	6.5%
Facet Injury		
none	94	34.2%
F1	26	9.5%
F2	42	15.3%
F3	14	5.1%
F4	99	36.0%
Modifier		
none	142	51.6%
1	55	20.0%
2	35	12.7%
3	35	12.7%
4	8	2.9%

**Table 3:** AO Spine injury morphology, treatment, and secondary dislocations.

Injury Type	Primary Stabilization	Patients (*n*)	Secondary Dislocations (*n*)	Secondary Dislocation Rate	Secondary Dislocation Rate per Injury Type
B2	anterior	16	2	12.5%	5.1%
	posterior	11	0	0.0%	
	combined	12	0	0.0%	
B3	anterior	64	2	3.1%	3.1%
	posterior	12	0	0.0%	
	combined	20	1	5.0%	
C	anterior	45	6	13.3%	4.3%
	posterior	20	0	0.0%	
	combined	75	0	0.0%	
total	anterior	125	10	8.0%	4.0%
	posterior	43	0	0.0%	
	combined	107	1	0.9%	

**Table 4:** Patient, injury, and treatment characteristics potentially favoring the occurrence of secondary dislocations in the total patient sample.

Potential Risk Factors	No Secondary Dislocation (*n* = 264)	Secondary Dislocation (*n* = 11)	*p*
Patient Characteristics			
Age [years, mean (SD)]	58.7 (20.0)	75.3 (7.6)	0.001 ^1^
Sex [% female]	25.8	45.5	0.134 ^2^
Preexisting spine pathology * [%]	12.1	27.3	0.121 ^2^
Injury Morphology			
AO Spine Injury Type [%]			0.696 ^3^
B2	14.0	18.2	
B3	35.2	27.3	
C	50.8	54.5	
Multilevel Primary Injury [%]	6.8	0.0	0.999 ^2^
Any Modifier [%]	48.5	45.5	0.999 ^2^
M1	20.5	9.1	0.699 ^2^
M2	12.9	9.1	0.999 ^2^
(Potentially) unstable Facet Injury [%]	54.9	90.9	0.020 ^2^
Treatment			
Primary stabilization [%]			0.010 ^3^
anterior	43.6	90.9	
posterior	16.3	0.0	
combined	40.2	9.1	
Cervical collar postoperative ** [%]	92.0	100.0	0.999 ^2^

Statistical tests used were as follows: ^1^ Mann–Whitney U, ^2^ Fischer–Boschloo, ^3^ Fischer–Freeman–Halton. * Corresponding to Modifier M3. ** Soft or rigid cervical collar.

**Table 5:** Patient, injury, and treatment characteristics potentially favoring the occurrence of secondary dislocations in patients with primary solitary anterior stabilization.

Potential Risk Factors	No Secondary Dislocation (*n* = 115)	Secondary Dislocation (*n* = 10)	*p*
Patient Characteristics			
Age [years, mean (SD)]	55.9 (20.0)	71.1 (7.9)	0.002 ^1^
Sex [% female]	4.3	50.0	0.088 ^2^
Preexisting spine pathology * [%]	3.5	20.0	0.073 ^2^
Injury Morphology			
AO Spine Injury Type [%]			0.096 ^3^
B2	12.2	20.0	
B3	53.9	20.0	
C	33.9	60.0	
Multilevel Primary Injury [%]	7.8	0.0	0.999 ^2^
Any Modifier [%]	52.2	40.0	0.524 ^2^
M1	31.3	10.0	0.279 ^2^
M2	14.8	10.0	0.999 ^2^
(Potentially) unstable Facet Injury [%]	40.0	90.0	0.005 ^2^
Treatment			
Bone grafting [%]	53.0	80.0	0.183 ^2^
Cervical collar postoperative ** [%]	94.8	100.0	0.999 ^2^

Statistical tests used were as follows: ^1^ Mann–Whitney U, ^2^ Fischer–Boschloo, ^3^ Fischer–Freeman–Halton. * Corresponding to Modifier M3. ** Soft or rigid cervical collar.

**Table 6:** Patient characteristics and injury morphology of cases with secondary dislocations and treatment of secondary dislocation.

No	Sex	Age	Level	Injury Type	Primary Treatment	Therapy of Secondary Dislocation
1	f	91	C6/7	B2	ACDF C6/7 with plate and iliac crest graft	Posterior instrumentation C5/6/7/Th1
2	f	74	C5/6	B2	ACDF C5/6 with plate and iliac crest graft	Removal of plate and ACDF C5/6/7 with plate
3	m	78	C6/7	B3	Posterior instrumentation C3/4 to C6/7 andAnterior fixation C5 to C7 with plate	Posterior instrumentation C3/4/5/6/7Anterior fixation C5 to C7 with plate
4	m	82	C5/6	B3	ACDF C5/6 with plate and iliac crest graft	Posterior instrumentation C3/4/5/6/7 and ACDF C4/5/6/7 with plate and intervertebral cages
5	m	66	C6/7	B3	ACDF C5/6/7 with plate and iliac crest grafts	Halo fixator
6	f	80	C6/7	C	ACDF C6/7 with plate and allogenous bone graft	Posterior instrumentation C4/5 to Th1/2
7	f	70	C6/7	C	ACDF C6/7 with plate and iliac crest graft	Posterior instrumentation C3/4/5 to Th1/2
8	m	68	C6/7	C	ACDF C6/7 with plate and iliac crest graft	Posterior instrumentation C6/7/Th1/2
9	f	74	C6/7	C	ACDF C5/6/7 with plate and intervertebral cages	Posterior instrumentation C5/6/7/Th1
10	m	79	C6/7	C	ACDF C6/7 with plate and iliac crest graft	Posterior instrumentation recommended; patient refused
11	f	67	C5/6	C	ACDF C5/6 with plate and intervertebral cage	Posterior instrumentation C4/5/6/7

f, female; m, male; ACDF, anterior cervical discectomy and fusion. Patients 3, 6, and 11 corresponding to Figure 2, Figure 3 and Figure 4.

## Data Availability

All data and materials regarding this study are available from the corresponding author on reasonable request.

## Supplementary Materials

The following supporting information can be downloaded at: https://www.mdpi.com/article/10.3390/jcm13030700/s1, Table S1: Comparison of demographic variables and injury morphology between treatment groups.; Table S2: Frequency of primary stabilization methods (anterior, posterior, combined) in different age groups (years) depending on AO Spine primary injury morphology.
