# Supplementary material for: Secondary Dislocations in Type B and C Injuries of the Subaxial Cervical Spine: Risk Factors and Treatment
Source: J Clin Med. 2024 Jan 25;13(3):700. doi: 10.3390/jcm13030700 (PMC10856098; doi:10.3390/jcm13030700)
Supplement: Supplementary file 1 [file jcm-13-00700-s001.zip › Supplementary Table S1.pdf]

Supplementary Table S1: Comparison of demographic variables and injury morphology between treatment groups.

Variable	Anterior (n = 125)	Posterior (n = 43)	Combined (n = 107)	p
**Patient Characteristics**				
Age [years, mean (SD)]	57.4 (19.8)	70.1 (16.6)	57.3 (20.0)	**<.001**[1]
Sex [% female]	24.0	34.9	26.2	.357[2]
Preexisting spine pathology [%]	4.8	32.6	14.0	**.023**[2]
**Injury Morphology**				
AO Spine Injury Type [%]				**<.001**[3]
B2	12.8	25.6	11.2	
B3	51.2	27.9	18.7	
C	36.0	46.5	70.1	
Multilevel Primary Injury [%]	7.2	4.7	6.5	.947[2]
Any Modifier [%]	51.2	62.8	39.3	**.023**[2]
(Potentially) unstable Facet Injury [%]	44.0	58.1	70.1	**<.001**[2]

Statistical tests used: [1]ANOVA, [2]Fischer Freeman Halton, [3]Chi^2.
