# Supplementary material for: Secondary Dislocations in Type B and C Injuries of the Subaxial Cervical Spine: Risk Factors and Treatment
Source: J Clin Med. 2024 Jan 25;13(3):700. doi: 10.3390/jcm13030700 (PMC10856098; doi:10.3390/jcm13030700)
Supplement: Supplementary file 1 [file jcm-13-00700-s001.zip › Supplementary Table S2.pdf]

Supplementary Table S2: Frequency of primary stabilization methods (anterior, posterior, combined) in different age groups (years) depending on AO Spine primary injury morphology.

			Primary stabilization			
**Age group**	**AOS Primary**		**anterioir**	**posterior**	**combined**	**total**
≤50	B2	Count	10	2	6	18
		% within row	55.6 %	11.1 %	33.3 %	100.0 %
	B3	Count	14	0	2	16
		% within row	87.5 %	0.0 %	12.5 %	100.0 %
	C	Count	19	3	32	54
		% within row	35.2 %	5.6 %	59.3 %	100.0 %
	Total	Count	43	5	40	88
		% within row	48.9 %	5.7 %	45.5 %	100.0 %
51-65	B2	Count	2	1	4	7
		% within row	28.6 %	14.3 %	57.1 %	100.0 %
	B3	Count	18	2	6	26
		% within row	69.2 %	7.7 %	23.1 %	100.0 %
	C	Count	11	5	14	30
		% within row	36.7 %	16.7 %	46.7 %	100.0 %
	Total	Count	31	8	24	63
		% within row	49.2 %	12.7 %	38.1 %	100.0 %
66-80	B2	Count	2	5	1	8
		% within row	25.0 %	62.5 %	12.5 %	100.0 %
	B3	Count	22	5	11	38
		% within row	57.9 %	13.2 %	28.9 %	100.0 %
	C	Count	12	9	23	44
		% within row	27.3 %	20.5 %	52.3 %	100.0 %
	Total	Count	36	19	35	90
		% within row	40.0 %	21.1 %	38.9 %	100.0 %
≥80	B2	Count	2	3	1	6
		% within row	33.3 %	50.0 %	16.7 %	100.0 %
	B3	Count	10	5	1	16
		% within row	62.5 %	31.3 %	6.3 %	100.0 %
	C	Count	3	3	6	12
		% within row	25.0 %	25.0 %	50.0 %	100.0 %
	Total	Count	15	11	8	34
		% within row	44.1 %	32.4 %	23.5 %	100.0 %
Total	B2	Count	16	11	12	39
		% within row	41.0 %	28.2 %	30.8 %	100.0 %
	B3	Count	64	12	20	96
		% within row	66.7 %	12.5 %	20.8 %	100.0 %
	C	Count	45	20	75	140
		% within row	32.1 %	14.3 %	53.6 %	100.0 %
	Total	Count	125	43	107	275
		% within row	45.5 %	15.6 %	38.9 %	100.0 %
